# Naturally-Derived Biphasic Calcium Phosphates through Increased Phosphorus-Based Reagent Amounts for Biomedical Applications

**DOI:** 10.3390/ma12030381

**Published:** 2019-01-25

**Authors:** Aura-Cătălina Mocanu, George E. Stan, Andreea Maidaniuc, Marian Miculescu, Iulian Vasile Antoniac, Robert-Cătălin Ciocoiu, Ștefan Ioan Voicu, Valentina Mitran, Anișoara Cîmpean, Florin Miculescu

**Affiliations:** 1 Department of Metallic Materials Science, Physical Metallurgy, University Politehnica of Bucharest, 313 Splaiul Independentei, J Building, District 6, 060042 Bucharest, Romania; mcn_aura@hotmail.com (A.-C.M.); andreea.maidaniuc@gmail.com (A.M.); m_miculescu@yahoo.com (M.M.); antoniac.iulian@gmail.com (I.V.A.); ciocoiurobert@gmail.com (R.-C.C.); 2 Department of Research, Development and Innovation, S.C. Nuclear NDT Research & Services S.R.L, 104 Berceni Str., Central Laboratory Building, District 4, 041919 Bucharest, Romania; 3 National Institute of Materials Physics, Laboratory of Multifunctional Materials and Structures, 405A Atomistilor Str., 077125 Măgurele-Ilfov, Romania; 4 Destructive and Nondestructive Testing Laboratory, S.C. Nuclear NDT Research & Services S.R.L, 104 Berceni Str., Central Laboratory Building, District 4, 041919 Bucharest, Romania; 5 Department of Analytical Chemistry and Environmental Engineering, University Politehnica of Bucharest, 1-7 Gh. Polizu Str., Polizu campus, L 015 Building, District 1, 011061 Bucharest, Romania; svoicu@gmail.com; 6 Department of Biochemistry and Molecular Biology, University of Bucharest, 91-95 Splaiul Independentei, 050095 Bucharest, Romania; valentinamitran@yahoo.com (V.M.); anisoara.cimpean@bio.unibuc.ro (A.C.)

**Keywords:** dolomitic marble, seashell, CaCO_3_ derived-calcium phosphates, modulated synthesis set-up, SEM, image analysis, pre-osteoblasts

## Abstract

Calcium carbonate from marble and seashells is an eco-friendly, sustainable, and largely available bioresource for producing natural bone-like calcium phosphates (CaPs). Based on three main objectives, this research targeted the: (i) adaptation of an indirect synthesis route by modulating the amount of phosphorus used in the chemical reaction, (ii) comprehensive structural, morphological, and surface characterization, and (iii) biocompatibility assessment of the synthesized powdered samples. The morphological characterization was performed on digitally processed scanning electron microscopy (SEM) images. The complementary 3D image augmentation of SEM results also allowed the quantification of roughness parameters. The results revealed that both morphology and roughness were modulated through the induced variation of the synthesis parameters. Structural investigation of the samples was performed by Fourier transform infrared spectroscopy and X-ray diffraction. Depending on the phosphorus amount from the chemical reaction, the structural studies revealed the formation of biphasic CaPs based on hydroxyapatite/brushite or brushite/monetite. The in vitro assessment of the powdered samples demonstrated their capacity to support MC3T3-E1 pre-osteoblast viability and proliferation at comparable levels to the negative cytotoxicity control and the reference material (commercial hydroxyapatite). Therefore, these samples hold great promise for biomedical applications.

## 1. Introduction

Orthopedic surgery advancements outlined a new, challenging, and necessary era for bone-loss reconstruction. Apart from small skeletal fractures when bone can repair itself, extensive bone defects that are above the critical size and result from accidents, trauma impact, or bone diseases, require filling treatment techniques [1,2,3,4].

Currently, the standard technique for bone reconstruction involves bone tissue harvesting from different body parts of the patient, process known as autografting/autologous bone grafting [5,6]. This method has several downsides including morbidity, supplementary surgery and reduced bone graft quantities [7,8], and therefore, is incompatible for massive bone defects repair. As an alternative, various synthetic grafts, including calcium phosphate-based materials, stirred up interest for almost three decades due to their biocompatibility and osteoconductivity properties [5,9,10]. Now, along with the high demand for reconstruction materials and the technological advancements which allow the industrial production of biomimetic calcium phosphates (CaPs), research aims turned to the bio-functionalization of natural resources (seashells, bovine bone, marble) as an environmentally friendly, sustainable and cost-effective alternative [11,12,13,14,15,16,17,18].

It is stated that an ideal biomaterial destined for skeletal repair applications should mimic the biological, compositional, and mechanical properties of the host bone and also create the necessary niche for further functionalization [1,19,20]. This is the reason why recent studies were focused on the synthesis of hydroxyapatite (HA) and acidic CaPs cements consisting of brushite (DCPD) and monetite (DCPA)—an anhydrous form of brushite [21,22,23], naturally found in bone, teeth, and renal calculi [24,25]. All three-candidate materials are crystalline forms of calcium phosphates. Hydroxyapatite has been widely reported as the preferred bone grafting material, and brushite and monetite were involved mainly in bone cement preparation due to their resorbable, self-regenerating, and osteoconductive character [1,25]. Under physiological conditions, brushite is metastable and highly reactive and was shown to reprecipitate into hydroxyapatite [21]. In addition, it is less soluble and forms first throughout cement reactions, even though monetite is a more stable phase [5,26,27]. Recent in vivo results, obtained after monetite-based granules implantation, showed an improved degradation and bone regeneration than the hydroxyapatite-based ones [23].

The morphological aspects such as texture, roughness and topographic patterns stand as well as essential factors for the biological success of ceramic materials/ceramic materials-based structures [28,29,30]. Along with chemical composition, surface features dictate the cellular behavior in terms of adhesion, differentiation, migration and proliferation (both in vitro and in vivo), and the degree of bone formation [19,31,32,33,34,35,36]. 

It was observed that a micrometric texture consisting of alternate valleys and peaks is relevant for the cells cytoskeleton organization [31,34,37]. Currently, 3D digital topographic reconstruction based on morphological analysis can provide enhanced insight on surface texture and topographic patterns. Also, it allows for quantification of surface roughness parameters which are classified as amplitude, spatial, and hybrid parameters [34,38,39]. A rather moderate rough surface with microporosity or grooves promotes in vivo the biological mediators secretion, which leads to cell adhesion and migration and new bone matrix formation [37,40]. It was reported that a depth of 2–5 nm on the structure’s surface is required for material–cell interaction, attachment, and development [37].

Most CaPs inherit an osteoconductive behavior due to their surface features. Biodegradable forms such as brushite and monetite are difficult to investigate in terms of surface topography influence on resorption mechanism, over a prolonged period of time due to ionic release in the in vitro culture medium [1,27].

Given the high frequency of orthopedic problems, we aimed for a resolution based on sustainable raw resources (marble and seashells) for a facile, cost-efficient, and direct synthesis (with less intermediary technological stages) of biomaterials with biological potential at least similar with that of commercial synthetic available materials. We followed this route to induce directly the synthesis of biphasic calcium phosphates compounds. Therefore, the main purpose of this research study resides on the development of novel naturally-derived biphasic CaPs with different phase composition, based on incipient results previously reported [11,12]. Further, the attention falls on the assessment of the adequate necessary reagent amount ranges, capable to allow for the synthesis of HA/DCPD and DCPD/DCPA biphasic materials through the developed methodology. This involved an adapted synthesis set-up [12] based on the conversion of natural calcium carbonate (CaCO_3_) precursors to biomimetic calcium phosphates (CaPs). An extensive characterization of the raw materials (i.e. marble and seashell) was presented in a previously published article [11]. This article further investigates the chemical reactions dynamics induced by the variation of phosphoric acid amount involved in the synthesis reaction. The modulation involved the gradually increase of phosphoric acid quantity starting with the stoichiometrically amount (considered as starting point of modulation −0%) up to 90% acid addition above the stoichiometry. Apart from a preliminary 0–30% reagent amount modulation [12], for which the new extended experimental sample set of investigations provided reproducible results, the key insights of this article rely on the further acid increment (40–90%). Thus, a complex characterization, focused on physico-chemically and digitally enhanced topographic features, was conducted along with an in vitro evaluation of the biological performance of all synthesized powdered samples in terms of pre-osteoblast viability and proliferation. 

## 2. Materials and Methods 

### 2.1. Ceramic Materials Synthesis 

Dolomitic marble and *Mytilus galloprovincialis* seashells were thermally treated at 1300 °C for 6 h for CaCO_3_ dissociation to calcium oxide (CaO). Previously reported investigations regarding the thermal transformations of both precursors [11] confirm that after the CO_2_ loss, the obtained CaO phase is stable at 1100–1200 °C and therefore the thermal treatment conducted at 1300 °C ensures a complete decomposition. The resulting powder was further hydrated with distilled water, filtered, deposited in thin layer on watch glass and dried for 144 hours at room temperature (RT) resulting the calcium hydroxide (Ca(OH)_2_) powder and no residual water [11]. Then, the Ca(OH)_2_ compound was weighed on a calibrated four decimal analytical balance (Kern & Sohn GmbH, Balingen, Germany) and treated with various amounts of phosphoric acid (H_3_PO_4_, Sigma-Aldrich, St. Louis, MO, USA). According to stoichiometrically calculated amounts (S.C.A.), 10 g of Ca(OH)_2_ were mixed with 200 mL distilled water and 5.5 mL of phosphoric acid, drop-wise added at a rate of 1 mL/min at room temperature (RT). The modulation of the products final chemical composition required a controlled addition of H_3_PO_4_ with respect to the S.C.A. ratio, incrementally increasing the acid volume in steps of 10% up to a maximum value of 90% (e.g. 0-M/S = S.C.A = 5.5 mL, 10-M/S = 5.5 mL + 10% × 5.5 mL = 6.05 mL, 20-M/S = 5.5 mL + 20% × 5.5 mL = 6.6 mL). Consequently, the Ca/P molar ratios of the solution were modified as indicated in Table 1, such to explore/enable the formation of other CaP-like phases than HA. The resulted slurries were stirred at 25 °C for 2 h, followed by aging for 72 h at RT, and drying at 100 °C for 2 h. The synthesized powders were deposited in Petri dishes and sealed in a desiccator. Further, the dried ceramic powders were ground in a planetary mill with agate balls, granulometric sorted with standardized sieves (<20–100 μm particle size) and transformed in cylindrical pressed samples (Φ 30 mm) by cold isostatic pressing with a force of 10 MPa. After 24 h at RT, pressed samples’ with parallel planar surfaces were obtained by polishing with P2500 grade abrasive sandpaper. 

For an easy tracking the sample codes included the amount of phosphoric acid added above the S.C.A, expressed in percents (0–90%) and the precursor’s abbreviation—M for marble; S for seashell (e.g., 20-M represents the sample prepared from marble in which a chemical treatment with S.C.A. + 20% phosphoric acid was used). 

For structural and cellular investigations, commercial hydroxyapatite (Merck KGaA, Darmstadt, Germany) was used as a reference material (Ref.). 

### 2.2. Characterization Techniques 

#### 2.2.1. XRD Analysis

The crystalline status and phase composition of the synthesized materials was investigated by X-ray diffraction (XRD) with a Bruker D8 Advance diffractometer (Bruker Corporation, Billerica, MA, USA) with Cu K_α_ (λ = 1.5418 Å) radiation, equipped with a Lynx Eye linear detector type, in Bragg–Brentano geometry. The samples were scanned in the 2θ angular range of 9–55° with a step size of 0.04° and 2 s acquisition time/step.

#### 2.2.2. FT-IR Spectroscopy Measurements

The analysis of the bonding architecture and identification of functional groups present in the samples was performed by Fourier transform infrared (FTIR) spectroscopy with a Perkin Elmer Spectrum BX II spectrometer (PerkinElmer, Inc., Waltham, MA, USA), in attenuated total reflectance (ATR) mode using a Pike-Miracle head with diamond-ZnSe crystal. The spectra were recorded in the range 4000–500 cm^−1^, at a resolution of 4 cm^−1^ and 32 scans/experiment.

#### 2.2.3. Morphological and Compositional Evaluation and 3D Image Augmentation

The morphological evaluation of the ceramic green bodies was performed by scanning electron microscopy (SEM) with a Philips XL 30 ESEM TMP microscope (FEI/Phillips, Hillsboro, OR, USA). Micrographs were acquired at an acceleration voltage of 25 kV and 10 mm working distance. SEM investigations were performed in five randomly chosen areas. Topographic reconstruction of pressed samples along with the quantification of the surface roughness parameters were possible through SEM 3D top view image analysis via MountainsMap software (Digital Surf, Besançon, France). Six of the most relevant roughness parameters for morphological surface texture were graphically displayed.

The compositional evaluation was performed with a portable X-ray fluorescence spectrometer (SPECTRO xSORT, Kleve, Germany). Synthesized samples were analyzed without further preparation and results are reported as average of three measurements/sample. 

#### 2.2.4. Biocompatibility Experiments

Indirect contact studies were performed using mouse pre-osteoblasts MC3T3-E1 (ATCC^®^, CRL-2593^TM^) grown in Dulbecco’s Modified Eagle’s Medium-DMEM (Sigma-Aldrich Co., St. Louis, MO, USA) supplemented with 10% fetal bovine serum (FBS) (Gibco (Life Technologies Corporation, Grand Island, NY, USA)) and 1% antibiotic-antimycotic mixture (Sigma-Aldrich Co., St. Louis, MO, USA). Marble- and seashell-derived powdered samples were sterilized for 1 h at 180 °C and afterwards subjected to extraction in culture medium at a concentration of 0.02 g/mL. The extracts obtained after incubation for 24 h at 37 °C were centrifuged, collected, and filtered using a filter with pore size 0.22 µm. 

The pre-osteoblasts were seeded at a density of 1 × 10^4^ cells cm^−2^ in a 96-well plate and incubated at 37 °C in a humidified atmosphere of 5% CO_2_/95% air for 24 h. After that, the cell culture medium was discarded and the cell monolayer was incubated for further 1 day and 3 days in 100 µL samples’ extracts. In parallel, the cells were incubated in DMEM containing 10% FBS without (cytotoxicity negative control) or with 5% dimethyl sulfoxide (DMSO) (Sigma-Aldrich Co., St. Louis, MO, USA) (cytotoxicity positive control). 

A qualitative cell viability analysis consisting of cell staining with LIVE/DEAD Cell Viability/Cytotoxicity Assay Kit (Molecular Probes, Eugene, OR, USA) was performed after 1 and 3 days of cell incubation in the samples’ extracts. The labeled cells were visualized using an inverted microscope equipped with epifluorescence (Olympus IX71, Olympus, Tokyo, Japan) and representative fields were captured with fluorescence imaging software Cell F. This assay was accompanied by a quantitative analysis of cell viability/proliferation, namely MTT [3-(4,5-dimethyl-2-thiazolyl)-2,5-diphenyl-2H-tetrazolium bromide) tetrazolium salt] assay, performed as previously described [41]. Briefly, cell monolayer was incubated with 1 mg mL^−1^ MTT solution for 3 h at 37 °C. The formazan produced by metabolically active viable cells was solubilized with DMSO and the absorbance of the dye was recorded at 550 nm using a microplate reader (Thermo Scientic Appliskan, Vantaa, Finland). 

## 3. Results and Discussion

### 3.1. Structural and Chemical Characterization

#### 3.1.1. XRD Analysis

**Figure 1:**
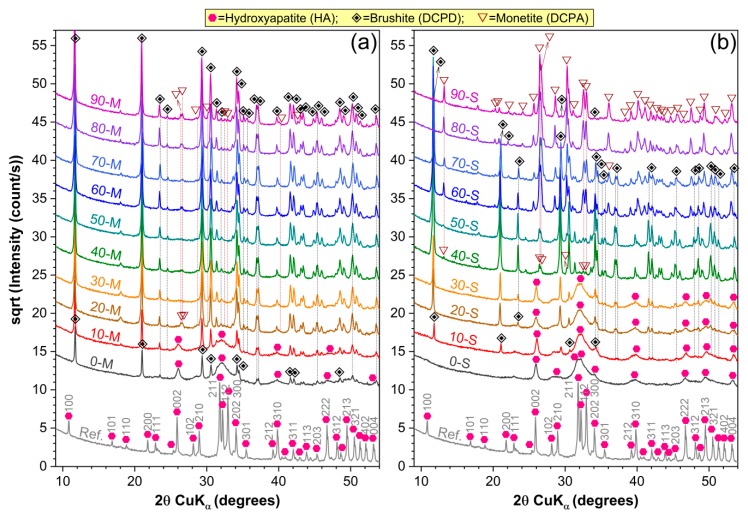
Comparative XRD patterns of the calcium phosphate powders synthesized using natural resources (i.e., (**a**) marble and (**b**) seashells) as calcium precursors and phosphoric acid amounts situated in the range 0–90%.

The XRD patterns of all the naturally synthesized ceramic powders are comparatively presented in Figure 1. A single phase material (i.e., monophasic HA) was obtained only in the case of stoichiometrically seashell-derived sample (0-S). The broad diffraction maxima (appertaining to a hexagonal HA, ICDD: 00-009-0432) with respect to the reference commercial HA material, indicates the nanostructured nature of the 0-S-type material. In the case of 0-M-type sample, the nano-sized HA is accompanied by a secondary dicalcium phosphate dehydrate (DCPD) monoclinic phase (CaHPO_4_·2H_2_O, brushite, ICDD: 01-072-1240).

Further acid addition strongly influenced the composition of the samples. It facilitated the formation of biphasic CaPs mixture—HA/DCPD for (0-10)-M and (10-30)-S samples. If in the case of M-derived samples (Figure 1a), the HA presence lingers up to 10%, for the S-derived samples (Figure 1b), the HA content progressively decreases for acid amounts in the range of 10–30%, such as at 40% it becomes extinct. Onward, the emergence of a dicalcium phosphate anhydrous (DCPA or monetite, CaHPO₄, ICDD: 01-070-1425) triclinic phase was signaled. Consequently, for higher acid additions (more than 10% and 30% in the case of M- and S-derived samples, respectively), the phase composition shifted from biphasic HA/DCPD to biphasic DCPD/DCPA. At higher acid increments, the DCPD/DCPA ratio remained seemingly similar for the M-type materials, contrary to the S-type ones, for which DCPA was found to predominate with acid additions situated over 60%. 

The coexistence of HA and DCPD is not unexpected, nor unprecedented. DCPD is more likely to precipitate in neutral or moderate acidic solutions at temperatures up to 40 °C as primary or secondary compound [42,43,44], and given the metastable thermodynamic character of the reaction, it is possible that in a saturated calcium and phosphate media the incipient HA crystals formation inhibited the precipitation and rapid development of DCPD crystals. It is also known that DCPD crystals can act as nuclei for HA evolution [45]. Nonetheless, the additional acid amount cannot only induce the abrupt (M-derived samples) or progressive (S-derived sample) reduction of HA content, but can promote DCPA formation based on its faster kinetic reaction [45,46]. In M-derived calcium carbonate, the natural Mg^2+^ dopant [11] stands as a possible inhibitory factor for HA precipitation even in stoichiometric conditions [42,47], and stabilized the biphasic mixture in acidic conditions. No unreacted Ca(OH)_2_ was transferred to final ceramic products structure, which indicated its complete conversion to CaPs.

#### 3.1.2. FTIR-ATR Measurements

**Figure 2:**
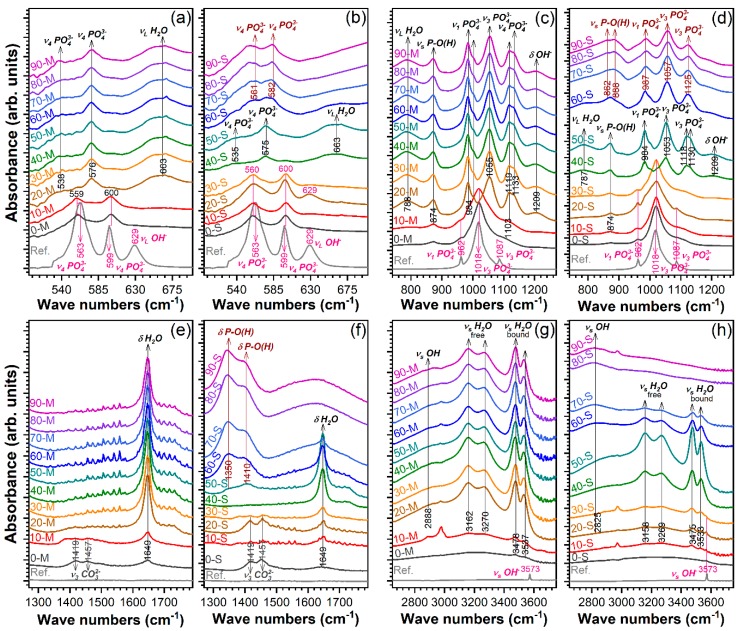
Comparative FTIR-ATR (attenuated total reflectance) spectra of the calcium phosphate powders synthesized using natural resources (i.e., (**a**,**c**,**e**,**g**) marble and (**b**,**d**,**f**,**h**) seashells) as calcium precursors and phosphoric acid amounts situated in the range 0–90%, collected in the four relevant wave numbers regions: (**a**,**b**) 700–500 cm^−1^; (**c**,**d**) 1300–700 cm^−1^; (**e**,**f**) 1800–1300 cm^−1^; and (**g**,**h**) 3750–2650 cm^−1^. To facilitate a better visual evaluation, the FTIR-ATR spectra were normalized to the intensity of the most prominent band region situated at ~1100–900 cm^−1^.

Figure 2 presents the FTIR-ATR comparative spectra for the products obtained using both type of calcium natural precursors and phosphoric acid addition in the range of 0–90%. 

The FTIR-ATR measurements confirmed the calcium phosphates formation and phase evolution previously disclosed by the XRD investigations. All the vibration bands characteristic to orthophosphate tetrahedral units into a hydroxyapatite-type structure were found to be prominent in the case of 0-M and 0-S samples: ν_4_ bending (~560–559 and ~600 cm^−1^) (Figure 2a,b), ν_1_ symmetric stretching (~962 cm^−1^) (Figure 2c,d), and ν_3_ asymmetric stretching (~1018 and ~1087 cm^−1^) (Figure 2c,d) [48]. To the difference of the pure, highly crystalline commercial HA, the spectra of the 0-M and 0-S samples elicited (i) broader peaks (testimony of their nanostructuring), (ii) the emergence of ν_2_ bending (~874 cm^−1^) and ν_3_ asymmetric stretching (~1419 and ~1457 cm^−1^) modes of carbonate groups (carbonatation of calcium phosphates synthesized under normal atmosphere conditions is to be expected), and (iii) a diminution of the vibration bands appertaining to structural hydroxyl units: libration (~629 cm^−1^) and stretching (~3573 cm^−1^) [48]. The higher than expected intensity of the (~874 cm^−1^ band (i.e. the intensity of ν_2_ (CO_3_)^2−^ should be one fifth of the ν_3_ (CO_3_)^2−^) ones [48]) recorded in the case of 0-M sample suggested the additive contribution of the vibration modes of acid phosphate [35,48], which emphasizes the concurrent formation of non-apatitic environments. This is in agreement with the XRD results (Figure 1a), which highlighted the simultaneous formation of DCPD along HA for the 0-M sample. In congruence with the XRD analyses, the FTIR-ATR results indicated that dramatic structural modifications occur at the same thresholds: 20-M, 40-S and 60-S. In the case of 20-M and 40-M samples, a series of newly emerged bands, characteristic to DCPD (brushite) [45,49,50,51,52,53] were emphasized: bending of (H–O)–P=O bonds in (HPO_4_)^2−^ (~538–535 and ~576–575 cm^−1^), H_2_O librations (~663 cm^−1^), P–O–H out-of-plane bending (~788–787 cm^−1^), stretching of P–O(H) bonds in (HPO_4_)^2^ molecules (~874 cm^−1^), ν_1_ symmetric stretching of phosphate (~984 and ~1004 cm^−1^), ν_3_ asymmetric stretching of (PO_4_)^3−^ (~1055–1053, ~1119–1118, and ~1133–1130 cm^−1^), (OH)^−^ in-plane bending (~1209 cm^−1^), H_2_O bending (~1649 cm^−1^), (P)O–H stretching (~2888 cm^−1^), two O–H doublet bands (~3162–3158 cm^−1^ and ~3270–3269 & ~3478–3475 and ~3537–3533 cm^−1^). The emergence of these two latter doublet bands represents a testimony for the presence of two types of water molecules in the structure of brushite [50,51]: the higher and the lower wavenumbers doublet bands appertaining to the bound and free water molecules, respectively. The issue is still disputed by spectroscopy specialists, the reverse assignment being sometimes endorsed [49,51]. The vibration bands typical for a DCPA (monetite)-type structure were clearly emphasized for the S-derived products starting with 60-S sample [49,50,51,52,53]: bending of O–P–O(H) bonds (~561 and ~582 cm^−1^), stretching of P–O(H) bonds in (HPO_4_)^2−^ molecules (~888 and ~862 cm^−1^), ν_1_ symmetric stretching of phosphate (~987 cm^−1^), ν_3_ asymmetric stretching of phosphate (~1057 and ~1125 cm^−1^), and in-plane bending of P–O(H) bonds (~1350 and ~1410 cm^−1^). The formation and development of DCPA is also marked by the extinction of the (OH)^−^ vibrations linked with the presence of water molecules present in the DCPD structure.

#### 3.1.3. XRF Evaluation. Ca/P Molar Ratio

The compositional evaluation of natural precursors (CaCO_3_) and both CaO and Ca(OH)_2_ powders is presented in Table 2 below. The samples consists initially of Ca, O and C. Results further revealed (i) a complete thermal dissociation of CaCO_3_ sustained by the absence of C content identified for CaO powder derived from both precursors [11] and (ii) minor concentrations of Mg^2+^ preserved along the thermal decomposition and hydration processes in case of marble precursor and marble derived CaO and Ca(OH)_2_ powders. Therefore, its influence during the synthesis process is still up for discussion, as described above. Regarding the calcium content from the calcium hydroxide derived from marble and the one derived from seashells, results revealed minor differences. This confirms our previously reported results [11].

**Figure 3:**
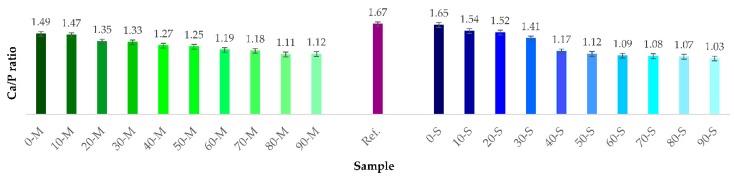
Ca/P molar ratio of the powders synthesized from both natural precursors (M—marble, S—seashell) with acid addition in the 0–90% range.

The XRF compositional evaluation performed on synthesized samples revealed that all investigated samples contain chemical elements characteristic to CaP phases: Ca, P, and O as major components, and the absence of other elemental traces. Ca/P molar ratios calculated on the basis of XRF results are graphically presented in Figure 3. The Ca/P values varied inversely proportional with the acid share used in the synthesis process, and their progressive decrease down to values situated in the vicinity of ~1, conferred further evidences of DCPD and DCPA phase formation. Compared to the reference sample, only 0-S sample elicited a Ca/P molar ratio close to the 1.67 theoretical value, specific to the stoichiometric hydroxyapatite [11,13,54].

### 3.2. Morphology and Roughness Evaluation

**Figure 4:**
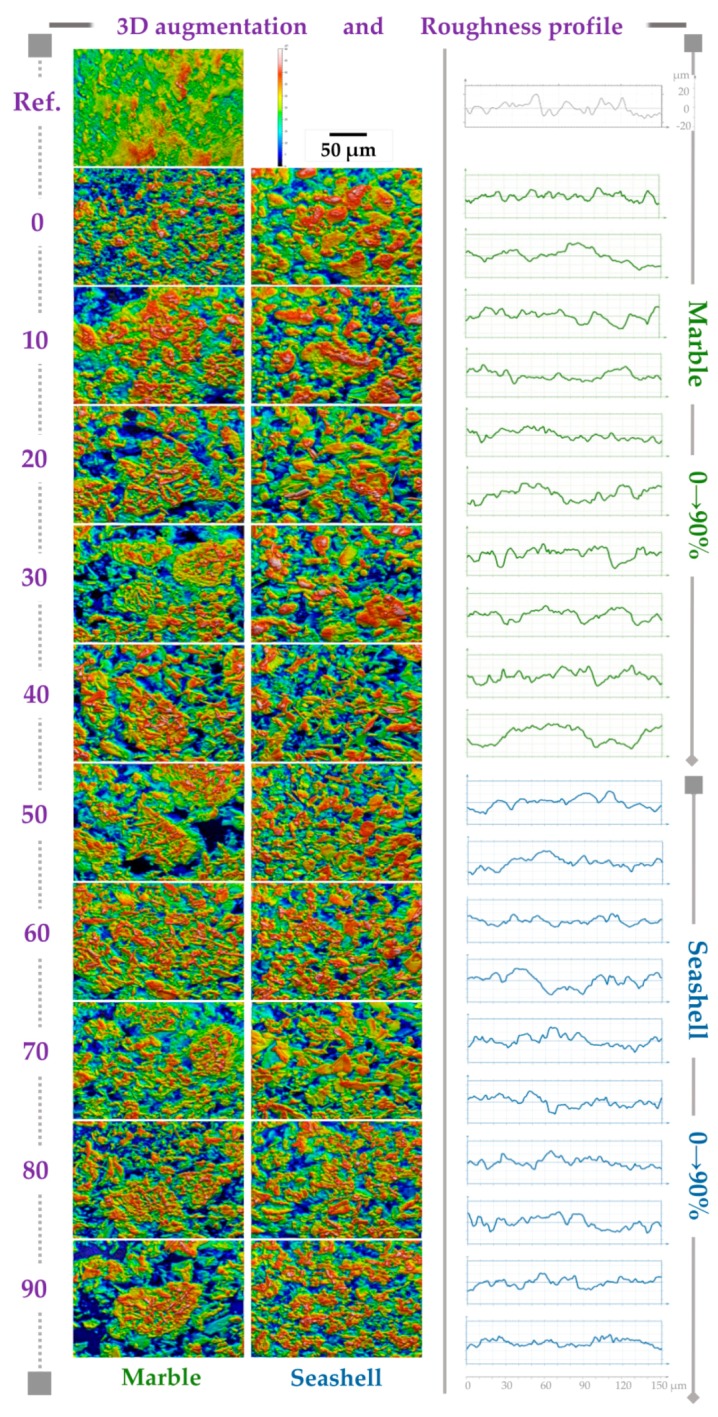
3D surface topology reconstruction on the basis of SEM micrographs processing via Mountains Map software and roughness profiles assessment. Images were recorded at 500× magnification. Top center image: Scale bar (50 µm) for all micrographs. For all roughness profiles, the scale bar is provided on the right side (up and down) of the figure.

**Figure 5:**
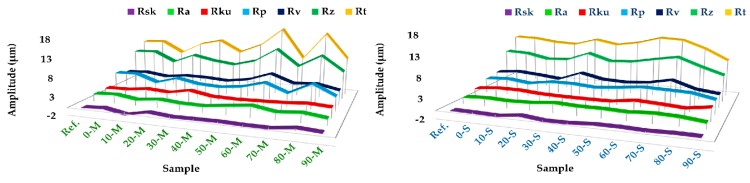
Roughness parameters quantification for marble (M) and seashell (S) derived samples: R_sk_—profile skewness, R_a_—profile arithmetic mean deviation, R_ku_—profile kurtosis, R_p_—maximum profile peak height, R_v_—maximum profile valley depth, R_z_—maximum height of profile, R_t_—total height of profile.

Digitally processed SEM images and their correspondent roughness profile are presented in Figure 4. For each 3D image, the following quantitative parameters were extracted from roughness profiles (Figure 5): R_sk_—profile skewness, R_a_—profile arithmetic mean deviation, R_ku_—profile kurtosis, R_p_—maximum profile peak height, R_v_—maximum profile valley depth, R_z_—maximum height of profile, R_t_—total height of profile. Their mathematical expressions are defined in ISO 4287:1997 [39].

Morphologically, the samples consisted of grains with different shapes and size distributions. An evolutionary tendency from well-defined polyhedral- to round-shaped grains, accompanied by a random and irregular grain size distribution with the increase of the acid amount, was observed. Initially, the grains conglomerate in larger, uniform, compact and centered isles (0–10%-M, 0–30%-S). Starting with 20%-M and 40%-S, the isles tend to disperse towards the corners of each morphological map until the 90% acid amount. Also, a shape transition from round grains/conglomerates to thin needle-like ones was evidenced up to 90% acid amount. 

Higher acid increments induced the formation of either mixed or distinctive isles formed from the two grain types. The morphology of the synthesized samples differed from the reference sample, which revealed uniform grain shapes and size distributions. 

The 3D reconstruction allowed for a colorimetric distinction of two areas: the navy blue and dark orange colors highlight the deepest valleys and highest mountain peaks. Their presence is strongly related to either the positive (mountains) or negative (valleys) values of R_sk_ parameter. The presence of valleys indicates the micro-porous character of the granular material, highly important for cell adhesion [34,39]. Both R_sk_ and R_ku_ parameters revealed a surface texture characterized by symmetry, sharpness and curvature of heights profile distributions [39]. According to the R_sk_ values (Figure 4), both M- and S-derived samples presented similar microporous patterns (>−1 μm), which is consistent with the profile valley depth (R_v_) values. Further, the R_ku_ graphic displayed as well cases of accentuated peak sharpness (R_ku_ > 3 μm) for 30-M and 50-S samples, which are associated with the starting points of the major phase composition shifts revealed by the XRD (Figure 1) and FTIR-ATR (Figure 2) investigations. At higher acid concentrations, the morphological assessments disclosed surfaces with predominantly uniform heights distribution and curvature, which correlates with the topological texture observed from 3D reconstruction images. 

Another important roughness parameter—R_a_—is related to the profile’s roughness amplitude and stochastic surface roughness. Slightly smoother surfaces were obtained for the S-derived samples (R_a_ values in the range ~1.3–2.5 μm), independent of the acid share used in the synthesis process. These results are endorsed by the total and maximum height profile—R_t_, R_p_, and R_z_—parameters: for a maximum peak height there is also a maximum valley depth, but graphically smoother. 

Overall, the morphological observations suggested that independent of the natural precursor and acid share, the powder products were characterized by a randomly distributed microporous surface texture with conglomerate isles. The morphological features are recognized as a key factor for both the in vitro and in vivo performance of biomaterials [28]. On a cellular level, the topographic surface parameters dictate cell adhesion and proliferation, along with their further proper functioning in what concerns the cytoskeletal organization and cell differentiation. These are directly related to the intracellular signaling mechanisms between cell receptors and material’s surface [34,37]. Therefore, a micro-scale texture coupled with a moderate surface roughness are the preferred characteristics for an appropriate cell anchorage and development. Also, one can expect that the conglomerate isles can act as center points for first cell-biomaterial contact and for further cell attachment, growth and proliferation. From this point of view, a suitable cellular behavior is anticipated for all synthesized bioceramic samples.

### 3.3. In Vitro Pre-Osteoblast Behavior

**Figure 6:**
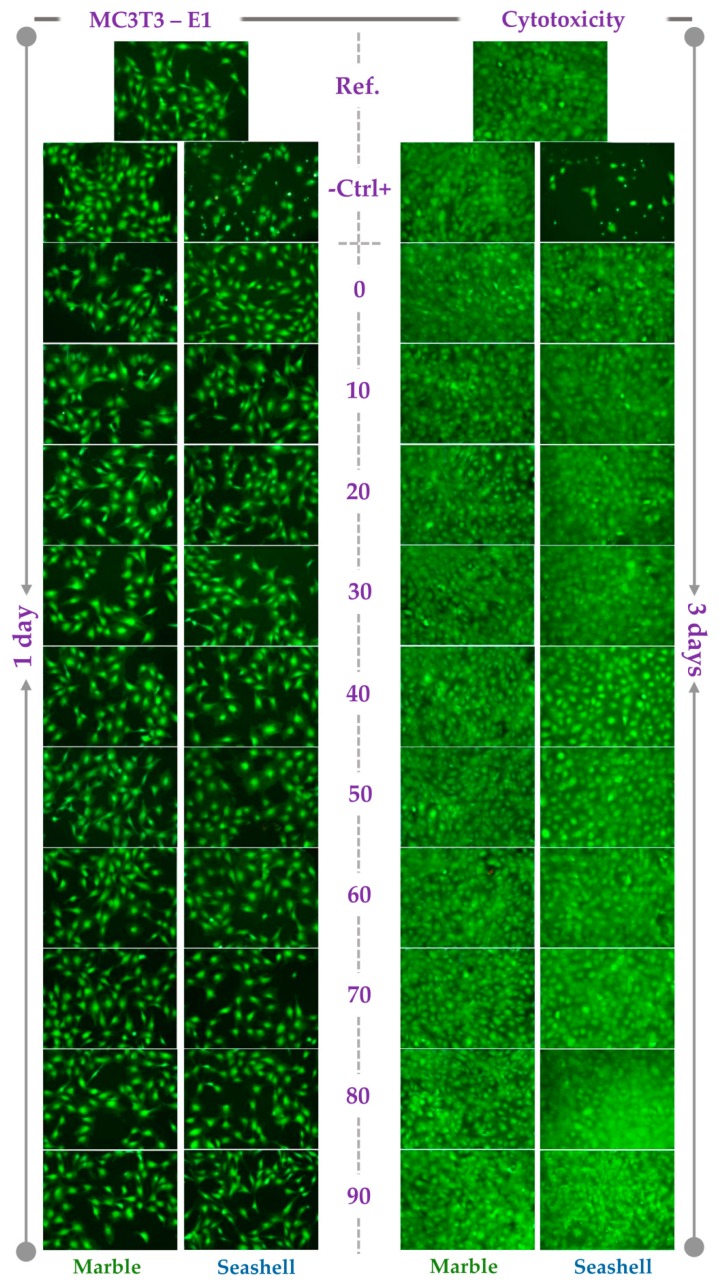
Fluorescence micrographs of the MC3T3-E1 pre-osteoblasts grown in the extracts of marble and seashell-derived powdered samples for 1 day and 3 days. Cell staining with the LIVE/DEAD Cell Viability/Cytotoxicity Assay Kit (green fluorescence: live cells; red fluorescence: dead cells). Scale bar: 100 µm.

The pre-osteoblast behavior in the extracts of the developed powdered samples was evaluated in terms of cell viability and proliferation by combining the results of the LIVE/DEAD Cell Viability/Cytotoxicity Qualitative assay and MTT assay. As shown in Figure 6, the extraction media of both M- and S-derived powdered samples sustained the cell viability regardless of acid increment. Thus, a high percentage of viable cells (green fluorescence) and a reduced number of dead cells (red fluorescence) were noticed at both incubation time points. Moreover, an increasing number of viable cells could be observed along the culture period suggesting the potential of these samples to support the pre-osteoblast proliferation. Likewise, typical healthy cell morphology was displayed except for the cytotoxicity positive control that exhibited mainly near-round cell shapes. The most probably, these cells are in progress of detachment from the underlying substrate as result of the toxicity elicited by DMSO. That also explains the progressive decrease in cell density along the culture period. Overall, the tested extracts showed cell morphologies and densities similar to the ones recorded for the reference sample extract and cytotoxicity negative control. Therefore, all analyzed powdered samples proved to be biocompatible. Interestingly, although no red-stained dead cells were noticed in case of (40–70)-S samples, lower cell densities were noticed. 

**Figure 7:**
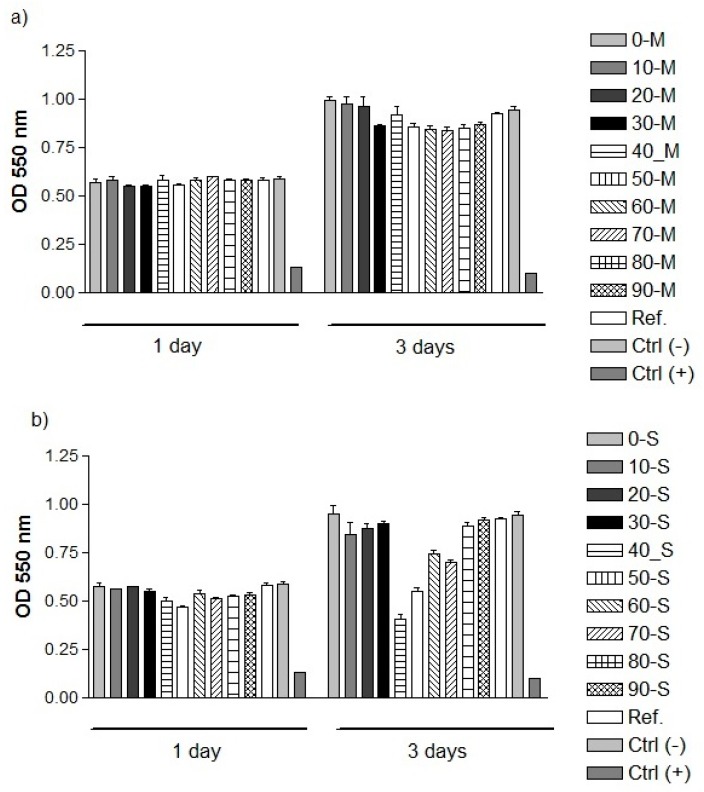
Cell viability/ proliferation of MC3T3-E1 pre-osteoblasts grown in the extraction media of marble- (**a**) and seashell- (**b**) derived powdered samples, as assessed by MTT assay (*n* = 3, mean ± SD).

Quantification by MTT assay of the cellular survival and proliferation capacity of MC3T3-E1 pre-osteoblasts demonstrated that the optical densities (OD) values, expressing the number of metabolically active viable cells, after 1 day of incubation in the extraction media of the analyzed M- and S-derived powdered samples were almost similar to the reference sample’s extract and cytotoxicity negative control (Figure 7). On the contrary, in the case of the cytotoxicity positive control, the number of viable cells was significantly reduced. The prolonged incubation of MC3T3-E1 cells (i.e. 3 days) led to higher OD values than those expressed at 1 day-time point with the exception of the 40-S extract that exhibited almost a similar number of metabolically active viable cells and the cytotoxicity positive control showing a decrease in OD values.

However, the fluorescence images acquired after performing the LIVE/DEAD Cell Viability/Cytotoxicity assay indicated that, except for the cytotoxicity positive control, all analyzed extracts exhibited higher cell densities at 3 days-time point than after 1 day of incubation. In comparison with the cytotoxicity negative control, reference sample and the extraction media of all other powdered samples, these findings conducted us to the conclusion that the lower OD values recorded at 3 days of cell incubation for (40–70)-S powdered samples could be rather due to the inhibition of the cell metabolic activity than to a decrease in cell viability and samples proliferation potential. Collectively, the results of the LIVE/DEAD Cell Viability/Cytotoxicity and MTT assays suggest that the extraction media of the M- and S-derived powdered samples exhibit good cytocompatibility and support the viability and proliferation of pre-osteoblast cells to a similar extent with the reference material extraction media and the cytotoxicity negative control.

## 4. Conclusions

This research study focused on the complex structural, morphological and biological characterization of a series of bioceramic samples derived from natural sources as dolomitic marble and seashells with respect to commercial highly crystalline hydroxyapatite. 

The adapted indirect synthesis route targeted the maximum additional phosphoric acid amount at which biomimetic biphasic CaPs can be obtained. The results demonstrated that biphasic hydroxyapatite/brushite mix is stable for 0–10% and 10–30% additional to stoichiometric acid amounts in the case of marble- and seashell-derived samples, respectively. Above these concentrations, the biphasic ceramic shifted to brushite/monetite mix, which demonstrated that a minimum of 20% and 40% additional acid amount is necessary for its precipitation. 

Since the synthesized materials are intended for reconstructive orthopedics, as bone fillers or cements, biological investigations coupled with digital topographic reconstruction were considered indispensable. The investigated roughness parameters revealed microporous surface textures independent of additional acid amounts or natural precursor. Further, indirect contact in vitro assessment of the MC3T3-E1 pre-osteoblast behavior proved that the extraction media of the powdered samples supported cell viability and proliferation at comparable levels to the ones recorded for the cytotoxicity negative control and commercial hydroxyapatite. The presented experimental approach could represent a first step towards the development of inexpensive yet promising biomedical solutions based on bioceramic biphasic powder systems with applications in bone regeneration.

## Figures and Tables

**Table 1:** Denomination of samples and Ca/P molar ratios of the precursor solutions.

H_3_PO_4_ Increment	0%	10%	20%	30%	40%	50%	60%	70%	80%	90%
**Sample Batch Code**	0-M; 0-S	10-M; 10-S	20-M; 20-S	30-M; 30-S	40-M; 40-S	50-M; 50-S	60-M; 60-S	70-M; 70-S	80-M; 80-S	90-M; 90-S
**Ca/P Molar Ratio**	1.67	~1.52	~1.39	~1.28	~1.19	~1.11	~1.04	~0.98	~0.93	~0.88

**Table 2:** XRF characterization of raw precursors and CaO, Ca(OH)_2_ powders.

Chemical Element (wt. %)		Ca	O	Mg	C
**Marble**	Raw precursor	33.30	42.86	0.83	22.71
	CaO	77.59	21.43	0.58	–
	Ca(OH)_2_	50.72	48.76	0.22	–
**Seashell**	Raw precursor	39.03	41.03	–	19.64
	CaO	75.06	24.54	–	–
	Ca(OH)_2_	51.21	48.39	–	–
